# Moldogenicity of dominant culturable molds in cigar tobacco leaves and GC-IMS–based analysis of volatile organic compounds

**DOI:** 10.3389/fmicb.2026.1843382

**Published:** 2026-08-28

**Authors:** Kuo Huang, Ge Zhang, Mengmeng Liu, Shaoxin Yang, Chen Chen, Beibei Zhu, Min Wei, Yong Pan, Xiaoyan Gao, Dong Li, Delong Xu, Fang Zheng, Fang Xue, Changwen Ye

**Affiliations:** 1 Zhengzhou Tobacco Research Institute, China National Tobacco Corporation, Zhengzhou, China; 2 College of Food Science and Technology, Huazhong Agricultural University, Wuhan, China; 3 College of Plant Protection, Southwest University, Chongqing, China; 4 China Tobacco Shandong Industrial Co., Ltd., Jinan, China; 5 Cigar Technology Innovation Center of China Tobacco, China Tobacco Sichuan Industrial Co., Ltd., Chengdu, China; 6 Technology Centre of Hubei China Tobacco Industry Co., Ltd., Wuhan, China

**Keywords:** cigar tobacco leaves, GC-IMS, moldogenicity, molds, VOCs

## Abstract

**Introduction:**

Cigar tobacco leaves (CTLs) are nutrient-rich and susceptible to mold contamination during fermentation and storage. This study aims to investigate the specific volatile profiles of dominant culturable molds recovered under the applied isolation conditions in CTLs and to establish a robust volatile organic compounds (VOCs)-based targeted monitoring strategy for preventing early-stage mold outbreaks.

**Methods:**

Eight dominant culturable molds were isolated from natural mold CTLs, and their spores were inoculated onto sterile CTLs. A combined visual scoring and mold counting system was utilized to evaluate the mold-inducing potential of the isolates. Gas chromatography-ion mobility spectrometry (GC-IMS) was employed to detect VOCs at the initial stage of visible mold onset. Furthermore, multivariate chemometric analyses, including principal component analysis (PCA) and partial least squares discriminant analysis (PLS-DA), were performed to process the data and screen for differential markers.

**Results:**

Based on the evaluation system, the isolates were quantitatively classified into high-moldogenicity (*Aspergillus montevidensis*, *Penicillium chrysogenum*, *Aspergillus pseudoglaucus*, and *Aspergillus versicolor*) and low-moldogenicity (*Talaromyces funiculosus*, *Trichoderma longibrachiatum*, *Aspergillus chevalieri*, and *Aspergillus sydowii*) strains. A total of 66 VOCs were identified by GC-IMS. PCA and PLS-DA not only clearly differentiated mold-contaminated samples from uninoculated controls but also clustered the strains according to their defined mold-inducing potential. Through strict variable screening (VIP > 1, *P* < 0.05), three VOCs—3-octanone (M), 2-pentanol, and 2-methyl-1-butanol—were identified as broad-spectrum early-warning markers for mold contamination. Additionally, 2-pentanol exhibited a distinctly higher relative abundance in high-moldogenicity strains, highlighting its potential as a quantitative indicator of infection severity.

**Conclusion:**

GC-IMS serves as a rapid and effective analytical tool for profiling dynamic VOC changes during fungal infection. The newly identified broad-spectrum markers and the quantitative classification model offer a promising targeted monitoring strategy to ensure safe cigar tobacco production.

## 1 Introduction

Cigars, known for their robust flavor, strong aroma, and unique taste, are a traditional tobacco product made from fermented cigar tobacco leaves (CTLs) (Morris and Fiala, 2015; Viola et al., 2016; Zhu et al., 2024; Zhang C. et al., 2025). CTLs are hygroscopic and contain abundant organic acids, sugars, and nutrients, creating favorable conditions for mold growth during fermentation, storage, and aging processes (Mutasa et al., 1990; Zhao et al., 2022). Molds damage the tissue structure of CTLs, weakening the desirable aroma of the raw materials/finished products and instead producing an unpleasant musty odor, ultimately causing irreversible economic losses (Zhao et al., 2020; Kępińska-Pacelik and Biel, 2021; Wei et al., 2024; Yang et al., 2015; Zhang H. et al., 2026; Fu et al., 2025). Moreover, certain molds can produce toxic and carcinogenic mycotoxins, posing severe safety risks to consumers (Kępińska-Pacelik and Biel, 2021; Dey et al., 2022; Vesper and Wymer, 2016). However, because eliminating these mycotoxins once formed is extremely difficult in the tobacco industry, a proactive strategy must focus on the rapid detection of fungal metabolic activity—specifically through volatile organic compounds (VOCs)—before severe structural damage and massive toxin accumulation occur. Therefore, given the global emphasis on product quality and safety, it is crucial to systematically study the moldogenicity (the potential to induce mold) of cigar-affecting molds and their early-stage volatile signature.

Deteriorating molds are diverse but share common characteristics: a strong propagation capacity and various reproductive modes. Studies have reported 130 genera and 231 species of molds that are responsible for commodity deterioration. Among them, the dominant molds in tobacco products are *Aspergillus* and *Penicillium*, with the occasional involvement of *Trichoderma* and *Rhizoctonia*. Identifying the main moldogenic species is key to mold prevention and control (Zhang C. et al., 2025; Wei et al., 2024). Previous studies have indicated that a significant number of molds exist in CTLs, but not all of them are harmful; some may even enhance cigar quality (Zhao S. et al., 2024). With advancements in chromatographic and spectroscopic techniques, mold identification has shifted toward the analysis of VOCs produced during mold growth and metabolism (Laura et al., 2016; Ruchika et al., 2016). Currently, while methods like solid phase microextraction–gas chromatography–mass spectrometry (HS-SPME-GC-MS) and electronic noses are used (Zeng et al., 2023; Yin et al., 2022), they often require complex sample preconcentration or specific sensors. Gas chromatography–ion mobility spectrometry (GC-IMS) has emerged as a powerful alternative. It separates gas components chromatographically and ionizes them, generating signals based on molecular drift (Christmann et al., 2024). This method offers high data-acquisition rates, repeatability, and minimal sample preparation (Joscha et al., 2022). GC-IMS has been successfully used in tobacco research; for example, Yan et al. (2022), Qin et al. (2021) employed it to distinguish the origin and grades of CTLs. Regarding mold detection, Yu et al. (2023) coupled GC-IMS with chemometrics to analyze early mold markers in naturally contaminated cigars, identifying generic markers such as 1-octen-3-ol. However, these previous studies largely treated the naturally contaminated microbiome as a “black box.” They primarily analyzed VOC samples without clarifying which specific fungal strains were responsible for the observed VOC changes, nor did they evaluate how different strains varied in their destructive potential.

To address these unresolved microbiological questions, this study proposes a novel strain-level evaluation model. Specifically, eight dominant culturable molds were first isolated from naturally moldy CTLs. We established a quantitative evaluation system to classify the moldogenicity of each specific strain based on visual scoring and mold counts. Subsequently, to avoid the confounding factors of mixed natural infections, sterile CTLs were inoculated with pure culture of these individual strains. Although fungal infection is a highly dynamic process requiring continuous time-series tracking for a true early-warning system, characterizing the strain specific VOC profiles at the initial stages of visible mold onset serves as a critical first step. By linking the chemical VOC data obtained via GC-IMS with the biological moldogenicity phenotypes, this study aims to screen for broad-spectrum mold markers and identify specific volatile signatures associated with highly destructive strains. Ultimately, these findings will expand the boundaries of industrial application by providing a precise, strain-resolved theoretical basis for early-stage mold monitoring and prevention in cigar tobacco.

## 2 Materials and methods

### 2.1 Collection of moldy CTL samples

Following the 5-point 3-layer sampling method, we collected moldy CTL samples (named moldy CTL 1) during the processing of Xue Zhi Meng No. 10 cigars (a commercial blended cigar product with a moisture content of 16%–17%) in China Tobacco Hubei Industrial Co., Ltd. (China) as well as moldy CTL samples at the pre-fermentation and fermentation stages (named moldy CTL 2 and moldy CTL 3, respectively; tobacco variety: Dexue No. 2, with a moisture content of 20%–23%) of Great Wall No. 3 cigar processing in China Tobacco Sichuan Industrial Co., Ltd. (China). Sampling was conducted on 4 April 2023, for moldy CTL 1 and 8 September 2022, for moldy CTL 2 and moldy CTL-3. The moldy CTL samples were placed in sterile sampling bags (with each bag containing 2 kg of samples), transported to the laboratory using cryopreservation boxes, and stored in a −80 °C freezer.

### 2.2 Culture media and reagents

The culture media used for mold cultivation included Bengal Red Agar for mold enumeration and Potato Dextrose Agar (PDA) for isolation and culturing. The reagents used were lactophenol cotton blue stain. Analytical-grade ortho-ketones, including 2-butanone, 2-pentanone, 2-hexanone, 2-heptanone, 2-octanone, and 2-nonanone, were purchased from Aladin Co., Ltd. (China) and used for VOC analysis.

### 2.3 Instruments and equipment

The isolation, purification, and preliminary identification of molds were performed using a sterile surgical table (BSC-1804IIA2, Suzhou Antai Airtech, China), a constant-temperature-and-humidity incubator (BXS-400S, Shanghai Boxun Medical Biological Instrument, China), an automatic colony counter (Scan1200, Interscience, France), a sterile homogenizer (BagMixer 400CC, Interscience, France), an optical microscope (CX41, Olympus Corporation, Japan), sterile homogenization bags, autoclaved scissors, autoclaved tweezers, glass stirring rods, 250 mL conical flasks, sealing films, a vortex oscillator, gradient dilution solutions, blood cell counting plates, coverslips, slides, and sterile gauze pads.

MALDI-TOF MS system: An Autof MS2000 system (Autobio, China) was used for fungal identification. GC-IMS system: A FlavourSpec system (G.A.S., Germany) equipped with a static headspace autosampler (CTC-PAL 3, CTC Analytics AG, Switzerland). The chromatographic column was an MXT-WAX GC capillary column (15 m × 0.53 mm × 1.0 μm), the column temperature was 60 °C, the analysis time was 30 min, and the carrier gas was N_2_ (purity ≥ 99.999%).

### 2.4 Mold isolation and purification

Moldy cigar tobacco leaf (CTL) samples were stored at −80 °C for approximately 2 weeks prior to analysis. Thawing was performed by placing the sealed sample bags at 4 °C for 12 h, followed by equilibration to room temperature for 30 min before processing.

Mold isolation and purification were performed according to GB 4789.15-2016. Briefly, 25 g of each moldy CTL sample was weighed and mixed with 225 mL of sterile water. The mixture was shaken thoroughly and diluted to prepare solutions with different concentrations (10^–2^, 10^–3^, and 10^–4^ g/mL). The diluted solutions were then inoculated onto Bengal Red Agar plates using the plate coating method. Four replicate plates were spread for each dilution. The plates were incubated at 28 °C for 3 days, and then the number of colonies in each plate was counted. Single colonies with a high abundance and distinct morphological characteristics were selected and purified to obtain purified strains. These strains were labeled as strains H-1, H-2, H-3, H-4, S-1, S-2, S-3, and S-4.

### 2.5 Mold identification

#### 2.5.1 Morphological evaluation

Mold morphological evaluation was performed according to GB 4789.16-2016. Briefly, the eight purified mold strains were inoculated at the center of eight different PDA plates and incubated at 28 °C for 5 days. Slides were prepared by sampling mycelia from the edge of each single colony, and the spore-producing structures were examined using an optical microscope.

#### 2.5.2 MALDI-TOF MS identification

In a biosafety cabinet, fungal mycelia or spores were scraped from PDA by using sterile pipette tips and placed into a centrifuge tube containing 1 mL of 75% ethanol. The sample was vortexed for 1 min to ensure thorough washing and then centrifuged at 13,000 rpm for 2 min. The supernatant was discarded, and the pellet was air-dried at room temperature for 5–10 min. Subsequently, 30–50 μL of filamentous fungal lysate was added to the pellet, which was then left undisturbed for 2–5 min before being vortexed for 2 min to ensure that the mycelia or spores lysed in the suspension. A 1 μL aliquot of the suspension was spotted onto the sample point of the MALDI-TOF MS target plate and allowed to dry naturally. Then, the target plate was inserted into the MALDI-TOF MS system, and batch identification was performed after editing the sample information. Instrument parameters: detector voltage 2.7 kV, repeller voltage 20.00 kV, extraction voltage 1.90 kV, focus voltage 7.00 kV, and mass scan range 2,000–20,000 Da. Matrix: α-cyano-4-hydroxycinnamic acid (CHCA). External calibration using ribosomal protein peaks from *E. coli* DH5α strain. Autobio Microbial Identification Database (V1.0). A score ≥ 9.0 (out of 10.0) was considered reliable identification at the species level.

#### 2.5.3 Molecular identification

Single fungal colonies growing on PDA were counted, and 20 mg of dried mycelia was ground into a powder by using liquid nitrogen. Then, DNA was extracted using the Ezup Column Fungal Genomic DNA Extraction Kit. The extracted DNA was used as a template for the PCR amplification of the ITS region by using primers ITS1 (5′-TCCGTAGGTGAACCTGCGG-3′) and ITS4 (5′-TCCTCCGCTTATTGATATATGC-3′) (Zhang G. et al., 2026). The PCR mixture (25 μL) contained 10× PCR buffer (2.5 μL), dNTPs (2.5 mmol/L each, 0.5 μL), the upstream primer (5 μmol/L, 1 μL), the downstream primer (5 μmol/L, 1 μL), Taq DNA polymerase (5 U/μL, 0.5 μL), sterile water (19 μL), and the DNA template (0.5 μL). The PCR program was as follows: pre-denaturation at 95 °C for 5 min; 30 cycles of denaturation at 94 °C for 30 s, annealing at 57 °C for 30 s, and extension at 72 °C for 90 s; and final extension at 72 °C for 10 min. The purified PCR products were sent to Sangon Biotech Co., Ltd (Shanghai, China) for sequencing. The resulting sequences were then compared with existing fungal ITS sequences in the NCBI database using the BLAST tool. The ITS sequences of the eight mold strains were deposited in the NCBI GenBank database under the accession numbers PZ742573 to PZ742580. A phylogenetic tree was constructed using MEGA11.0 software, employing the neighbor-joining method and a bootstrap value of 1,000.

### 2.6 Determination of the moldogenicity of dominant culturable molds in moldy CTLs

#### 2.6.1 Mold cultivation and spore suspension preparation

The eight dominant mold strains were inoculated onto PDA media. The plates were sealed and incubated in a constant temperature incubator at 28 °C for 5–7 days to ensure high spore viability. Following incubation, the mold plates were washed with sterile PBS containing 0.01% Tween-80 to prevent spore clumping. The resulting wash solution was filtered through four layers of sterile gauze to completely remove the mycelia. The filtrate was thoroughly homogenized by vortexing for 15–30 s, and the spores were counted using a hemocytometer. To ensure a comparable initial physical dose across all species, the eight mold spore suspensions were uniformly standardized by dilution to a concentration of 1.0 × 10^6^ spores/mL and stored at 4 °C for no more than 48 h before use. During inoculation, an appropriate amount of the standardized spore suspension was sprayed evenly onto the surface of 5.0 g of cigar tobacco leaves until the final leaf moisture content reached approximately 30% (Zhang G. et al., 2025). The leaves were then allowed to equilibrate at room temperature for 30 min before further incubation. Prior to inoculation, the healthy CTL substrates were subjected to high-pressure steam sterilization at 121 °C for 15 min. Although autoclaving may inevitably alter certain heat-sensitive chemical constituents and VOCs of the tobacco leaves, this step was essential to completely eliminate native background microorganisms, ensuring that any subsequent physical and chemical changes were exclusively attributable to the specifically inoculated mold strain. Two control (CK) groups were established: the CK1 group consisted of unsterilized CTLs inoculated with an equal volume of sterile PBS, while the CK2 group consisted of sterilized CTLs inoculated with an equal volume of sterile PBS. The subsequent microbiological analysis of the CK2 group (yielding 0.00 CFU/g) verified the complete efficacy of the sterilization process. Each sample was prepared in triplicate. The mold-inoculated CTLs and the control groups were placed into 250 mL Erlenmeyer flasks and sealed with Parafilm M to prevent microbial contamination. The flasks were placed in an incubator set at 28 °C and 70% RH for 5 days for an accelerated test (Zhang G. et al., 2025), and their positions were randomized to ensure uniform temperature exposure. After the 5 days period, the CTLs were assessed for mold growth.

#### 2.6.2 Mold content analysis

Mold-inoculated CTLs (5.0 g) were transferred into a sterile homogenization bag, followed by the addition of 45 mL of PBS (pH 7.2). The mixture was homogenized for 3 min using a stomacher. Serial 10-fold dilutions were prepared, and working dilutions of 10^–5^, 10^–6^, and 10^–7^ were selected for plating. A 0.1 mL aliquot of each dilution was spread onto Rose Bengal agar plates using the spread-plate method. Each dilution was plated in four technical replicates. The plates were incubated at 28 °C for 3–5 days. Only plates containing 15–300 CFU were counted. The mold content (CFU/g) was calculated using the following formula:


Moldcontent(CFU/g)=(AverageColonyCount×

DilutionFactor)/5.0g

The mold count data were log_10_-transformed prior to statistical analysis.

#### 2.6.3 Visual evaluation and moldogenicity classification

Seven experts visually evaluated mold development in mold-inoculated and control CTLs. Mold development was graded on a 6-point scale: Grade 0 (−, Score 0), Grade 1 (+, Score 1), Grade 2 (++, Score 2), Grade 3 (+++, Score 3), Grade 4 (++++, Score 4), and Grade 5 (+++++, Score 5). These grades corresponded to the severity levels of mold manifestation, with Grade 0 indicating no mold manifestation (mold coverage area of 0%–5%), Grade 1 indicating light mold manifestation (mold coverage area of 5%–20%), Grade 2 indicating mild mold manifestation (mold coverage area of 20%–40%), Grade 3 indicating moderate mold manifestation (mold coverage area of 40%–60%), Grade 4 indicating strong mold manifestation (mold coverage area of 60%–80%), and Grade 5 indicating severe mold manifestation (mold coverage area of 80%–100%). The visual evaluation was independently conducted by a panel of seven trained experts. The final grade for each sample was determined based on the average score.

To systematically evaluate the moldogenicity of the inoculated strains, a consistent two-tier classification rule was established using the unsterilized control (CK1) as a baseline. The CK1 group exhibited an intrinsic background mold count of approximately 10^6.23^ CFU/g and minimal visual mold development. The inoculated molds were classified into two groups: (1) high moldogenicity, defined by a mold count higher than CK1 (>10^6.23^ CFU/g) and a visual score of ≥Grade 4; and (2) low moldogenicity, defined by a mold count lower than CK1 (<10^6.23^ CFU/g) and a visual score of <Grade 4.

### 2.7 GC-IMS analysis of VOCs in CTLs

#### 2.7.1 Sample preparation

Mold-inoculated CTLs (1 g) were weighed and placed in a 20 mL headspace vial. Each sample was prepared in triplicate.

#### 2.7.2 GC-IMS analysis conditions

Headspace conditions: The incubation temperature was 80 °C, the incubation time was 15 min, the injection volume was 500 μL (with no split injection), the incubation speed was 500 rpm, and the injection needle temperature was 85 °C.

The carrier gas flow rate program was as follows: The initial flow rate was 2 mL/min, which was maintained for 2 min, followed by a linear increase to 10 mL/min within 8 min, a further increase to 100 mL/min within 10 min, and flow rate maintenance at 100 mL/min for 10 min. Retention indices (RIs) and drift times were normalized using external *n*-ketone standards and compared with database values.

Ion mobility spectrometry conditions: The ionization source was β-rays from a tritium radioactive source, the drift gas was high-purity N_2_ (purity ≥ 99.999%), the drift gas flow rate was 150 mL/min, the drift tube temperature was 45 °C, and the ionization mode was positive ion mode.

### 2.8 Statistical analysis

Data were analyzed using SPSS 22.0 and are expressed as mean ± standard deviation of three biological replicates. For mold count comparisons, log_10_-transformed CFU/g data were analyzed using ANOVA. When ANOVA indicated a significant difference (*P* < 0.05), Tukey’s HSD-test was applied for *post hoc* multiple comparisons. Homogeneity of variances was checked using Levene’s test, and normality was assessed using the Shapiro-Wilk test. All data met the assumptions for ANOVA.

For VOC data, raw GC-IMS spectra were processed using VOCal software (version 0.4.03). Compound identification was performed using the instrument’s built-in software, Laboratory Analytical Viewer (LAV), and the qualitative software GC × IMS Library Search, which integrates the NIST2020 and IMS databases. VOC fingerprints were constructed using the Reporter, Gallery, and Dynamic PCA plug-ins in LAV software, along with the GC × IMS Library Search. The identified VOCs were semi-quantitatively analyzed based on their normalized relative signal intensities (peak volumes). These peak volumes served as the data matrix for subsequent chemometric analyses. Principal Component Analysis (PCA) and Partial Least Squares Discriminant Analysis (PLS-DA) were performed using SIMCA software (version 14.0). Heatmap construction and hierarchical cluster analysis were performed using TBtools (version 2.0). A significance level of *P* < 0.05 was used for all statistical comparisons.

## 3 Results and discussion

### 3.1 Isolation, purification, and identification of dominant culturable molds in moldy CTLs

#### 3.1.1 Mold content analysis

**FIGURE 1:**
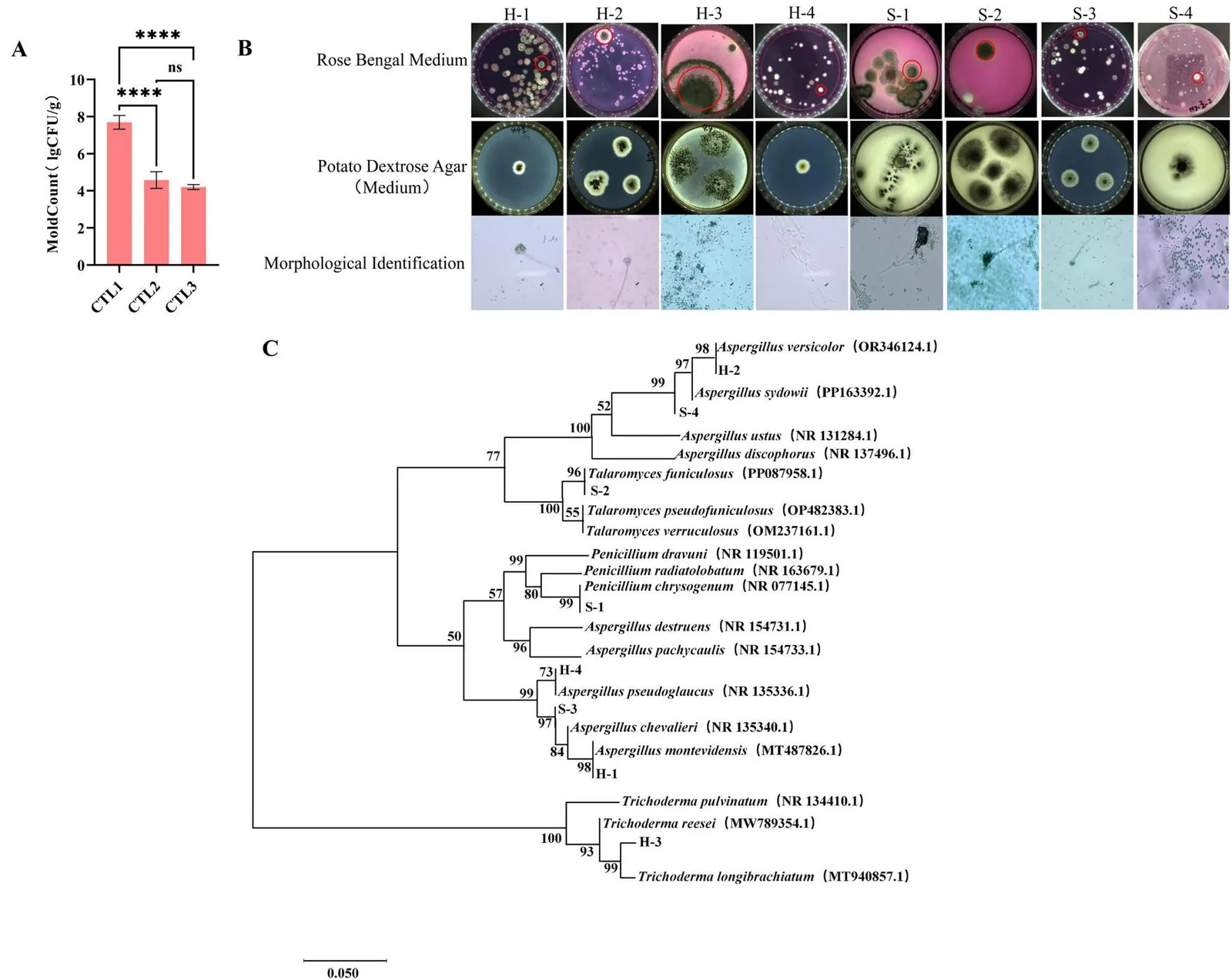
Isolation, purification, and identification of dominant culturable molds in moldy cigar tobacco leaves (CTLs). **(A)** Mold counts of CTL samples. **(B)** Colony morphologies and microscopic images of the eight purified mold strains grown on different media. **(C)** A phylogenetic evolutionary tree showing the evolutionary relationships of the eight identified molds. ****Indicates a statistically significant difference at *P* < 0.0001.

The mold counts of the moldy CTL samples (CTL 1, CTL 2, and CTL 3) are presented in Figure 1A. CTL 1 exhibited a significantly higher mold count compared to CTL 2 and CTL 3 (*P* < 0.0001). No significant difference was observed between the mold counts of CTL 2 and CTL 3, though both exceeded 10^4^ CFU/g. The abundance, diversity, and relative proportions of molds isolated on Rose Bengal agar plates are summarized in Table 1.

**TABLE 1:** Proportion of mold species in different moldy samples.

Group	Plate	Morphotype	No. of colonies (CFU)	Total CFU on plate	Proportion (%)	Assigned isolate ID
CTL1	1	1	91	247	36.84	H-1
	2	1	105		42.51	H-1
		2	9		3.64	H-2
	3	1	1		0.40	H-3
		2	2		0.81	S-2
	4	1	22		8.91	H-4
		2	17		6.89	S-3
CTL2	1	1	43	87	49.43	S-1
	2	1	10		11.49	S-3
		2	30		34.48	H-4
	3	1	1		1.15	H-3
	4	1	1		1.15	S-2
		2	2		2.30	S-4
CTL3	1	1	1	39	2.56	S-2
		2	2		5.13	S-4
	2	1	27		69.23	H-1
		2	5		12.82	S-4
	3	1	2		5.13	S-4
	4	1	1		2.56	H-3
		2	1		2.56	S-2

While the fungal profiles of the three samples groups showed both overlaps and variations, strains exhibiting identical colony morphologies were grouped together. Through this process, a total of eight dominant culturable mold strains were identified and selected for subsequent experiments based on their high prevalence and distinct morphological traits. Notably, strain H-1 was detected in both CTL 1 and CTL 3, accounting for 79.35% and 69.23% of the total counts, respectively, making it the absolute dominant mold. Strain H-2 was exclusively isolated from CTL 1 (3.64%), whereas strain H-3 was present across all three sample groups, albeit at lower proportions (0.40%, 1.15%, and 2.56%, respectively). Strain H-4 was found in CTL 1 (8.91%) and CTL 2 (34.48%).

Among the S-series strains, S-1 was isolated only from CTL 2 (49.43%). Strain S-2 was distributed across all three samples (CTL 1: 0.81%; CTL 2: 1.15%; CTL 3: 5.13%). Strain S-3 was identified in CTL 1 (6.89%) and CTL 2 (11.49%), while strain S-4 was isolated from CTL 2 (2.30%) and CTL 3 (23.08%).

#### 3.1.2 Isolation, purification, and identification of dominant culturable molds

The number and morphology of mold colonies growing on Bengal Red Agar and PDA plates were analyzed after 5 days of incubation. The colony morphologies of eight mold strains grown on the aforementioned plates are shown in Figure 1B.

The H-4 strain grew slowly on PDA, with its colonies exhibiting yellowish to yellow coloration on the surface and white to yellowish coloration on the reverse side. Its spores were spherical. In contrast, the H-3 strain demonstrated faster growth on PDA. Its colonies were green and surrounded by white mycelial bands, with its spores being oval or nearly spherical. The S-4 strain also grew slowly on PDA, forming fluffy colonies that were dark blue at the center and white at the edges, producing a dark brown exudate. Its conidial heads were spherical and radial. The H-2 strain exhibited slow growth on PDA, forming green fluffy or flocculent colonies. Dark green droplets were observed, and the conidial heads were spherical, hemispherical, or laxly radial. Similarly, the S-3 strain grew slowly on PDA, producing yellow colonies and spherical conidia. The H-1 strain exhibited slower growth on PDA, forming green colonies and spherical conidia. The S-1 strain grew slowly on PDA, forming irregularly shaped colonies with a green-to-gray-green surface. The conidia were spherical, ellipsoidal, or short columnar. Finally, the S-2 strain demonstrated slower growth on PDA, producing colonies with a gray-green, powdery, and slightly elevated surface. Its conidia were ellipsoid, and the spore-producing structures were broom-like and scattered outward.

According to the manufacturer’s guidelines for the MALDI-TOF MS identification kit, scores above 9.0 were reliable for identification at the species level, while scores between 6.0 and 8.9 were reliable for identification at the genus level; scores below 6.0 were considered unreliable for identification (Wolfram et al., 2019; Giebel et al., 2010). The MALDI-TOF MS identification results, including the detailed matching mass spectra and score rankings (Supplementary Figure 1) showed that strains S-1, S-2, S-3, H-1, H-2, H-3, and H-4 exhibited scores above 9.0. Therefore, these strains were reliably identified at the species level as *Penicillium chrysogenum* (*P. chrysogenum*), *Talaromyces funiculosus* (*T. funiculosus*), *Aspergillus chevalieri* (*A. chevalieri*), *Aspergillus montevidensis* (*A. montevidensis*), *Aspergillus versicolor* (*A. versicolor*), *Trichoderma longibrachiatum* (*T. longibrachiatum*), and *Aspergillus pseudoglaucus* (*A. pseudoglaucus*), respectively. The remaining strain, S-4, was identified as *Aspergillus sydowii* (*A. sydowii*), with a score between 6.0 and 9.0, indicating reliable identification at the genus level.

Homology analysis using the BLAST tool revealed that the ITS sequences of strains S-1, S-2, S-3, S-4, H-1, H-2, H-3, and H-4 were 100% identical to those of the following species: *P. chrysogenum* (NR 077145.1), *T. funiculosus* (PP087958.1), *A. chevalieri* (NR 135340.1), *A. sydowii* (PP163392.1), *A. montevidensis* (MT487826.1), *A. versicolor* (OR346124.1), *T. longibrachiatum* (MT940857.1), and *A. pseudoglaucus* (NR 135336.1), respectively. The phylogenetic tree depicted in Figure 1C further supported that the eight mold strains (S-1, S-2, S-3, S-4, H-1, H-2, H-3, and H-4) corresponded to *P. chrysogenum*, *T. funiculosus*, *A. chevalieri*, *A. sydowii*, *A. montevidensis*, *A. versicolor*, *T. longibrachiatum*, and *A. pseudoglaucus*. The MALDI-TOF MS identification results were consistent with the ITS-sequencing-based identification results, confirming the reliability of strain identification.

### 3.2 Analysis of the moldogenicity of dominant culturable molds

**FIGURE 2:**
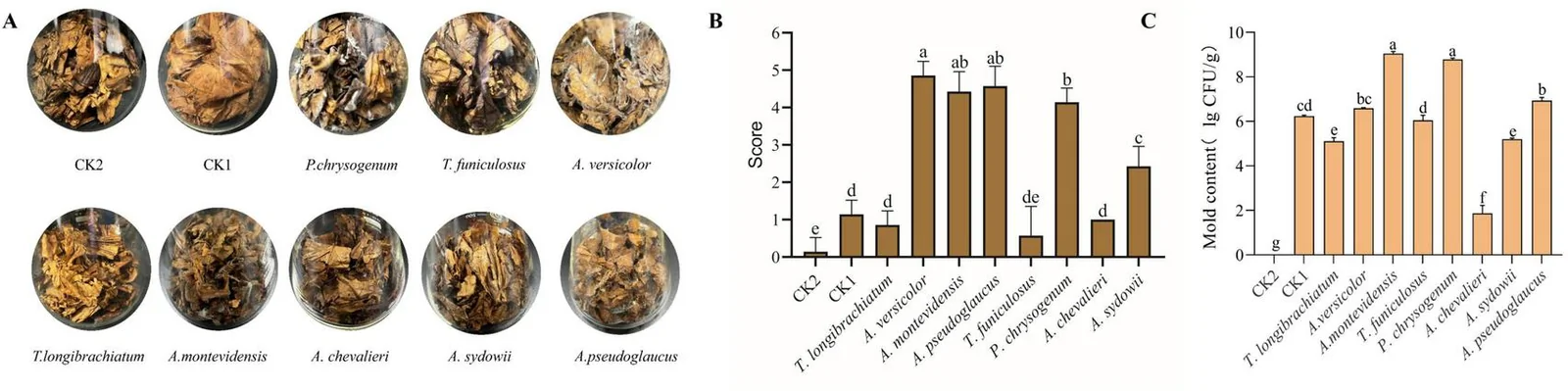
Moldogenicity evaluation in cigar tobacco leaves (CTLs) inoculated with different mold spore suspensions and incubated for 5 days. **(A)** Visual appearances; **(B)** visual evaluation of CTLs; **(C)** mold counts of CTLs. Different lowercase letters (a, b, c, etc.) above the bars indicate statistically significant differences between groups (*P* < 0.05) based on Tukey’s HSD test.

The moldogenicity of the eight dominant cultural molds isolated from moldy CTLs was evaluated after a 5-day inoculation period. The evaluation combined visual assessments and mold counts to establish a comprehensive classification (Figure 2).

#### 3.2.1 Visual evaluation of CTLs after inoculation

The visible mold manifestations and corresponding visual scores of the CTLs are presented in Figures 2A, B. The uninoculated sterilized control (CK2) maintained a clean appearance with a score near 0. The natural unsterilized control (CK1) exhibited slight natural fungal growth with a mean score of 1.14. Among the inoculated samples, CTLs inoculated with *A. versicolor*, *A. montevidensis*, *P. chrysogenum*, or *A. pseudoglaucus* exhibited severe mold manifestation, with mycelia densely covering the leaf surfaces, resulting in significantly high visual scores (Grade 4.0 or higher). In contrast, CTLs inoculated with *T. funiculosus*, *T. longibrachiatum*, *A. sydowii*, or *A. chevalieri* showed minimal to moderate mold development, with visual score below 4.0.

#### 3.2.2 Mold counts of CTLs after inoculation

To quantitatively validate the visual observations, the mold counts (log_10_ CFU/g) were analyzed (Figure 2C). The mold count of the CK2 group was close to the detection limit, confirming the efficacy of the initial sterilization. The baseline mold count in the CK1 group was 6.23 (log_10_ CFU/g). Strains including *A. montevidensis*, *P. chrysogenum*, *A. pseudoglaucus*, and *A. versicolor* exhibited exceptionally high proliferation capacities, reaching mold counts ranging from 6.56 to 9.04 (log_10_ CFU/g). Conversely, *T. funiculosus*, *T. longibrachiatum*, *A. sydowii*, or *A. chevalieri* displayed lower colonization efficiencies, with mold counts remaining below the CK1 baseline (ranging from 1.87 to 6.04 log_10_ CFU/g). Especially, *A. chevalieri* exhibited the lowest mold count, which aligned perfectly with the visual evaluation results.

#### 3.2.3 Comprehensive classification of moldogenicity

To establish a standardized and reproducible evaluation system, a classification matrix was developed using the CK1 baseline as the decision criterion. As detailed in Table 2, strains were strictly categorized into two groups: “High moldogenicity” (mold count > 6.23 log_10_ CFU/g and visual score ≥ 4.0) and “Low moldogenicity” (mold count < 6.23 log_10_ CFU/g and visual score ≤ 4.0). Based on this quantitative rule, *A. montevidensis*, *P. chrysogenum*, *A. pseudoglaucus*, and *A. versicolor* were robustly assigned to the high moldogenicity group, whereas *T. funiculosus*, *T. longibrachiatum*, *A. sydowii*, or *A. chevalieri* were classified into the low moldogenicity group. This clear categorization provided a reliable basis for the subsequent screening of mold-associated volatile markers.

**TABLE 2:** Classification of the eight dominant culturable molds based on visual scores and mold counts.

Strain/group	Mold count (log_10_ CFU/g)	Visual score	Decision criterion	Moldogenicity
CK1 (control)	6.23	1.14	Baseline	–
*A. montevidensis*	9.04	4.57	log_10_ mold count > 6.23; score ≥ 4.0	High
*P. chrysogenum*	8.79	4.14	log_10_ mold count > 6.23; score ≥ 4.0	High
*A. pseudoglaucus*	6.93	4.57	log_10_ mold count > 6.23; score ≥ 4.0	High
*A. versicolor*	6.59	4.86	log_10_ mold count > 6.23; score ≥ 4.0	High
*T. funiculosus*	6.04	0.57	log_10_ mold count < 6.23; score < 4.0	Low
*T. longibrachiatum*	5.10	0.86	log_10_ mold count < 6.23; score < 4.0	Low
*A. sydowii*	5.20	2.43	log_10_ mold count < 6.23; score < 4.0	Low
*A. chevalieri*	1.87	1.00	log_10_ mold count < 6.23; score < 4.0	Low

### 3.3 GC-IMS analysis of VOCs in CTLs after inoculation with different mold spore suspensions

#### 3.3.1 Spectral analysis of VOCs

**FIGURE 3:**
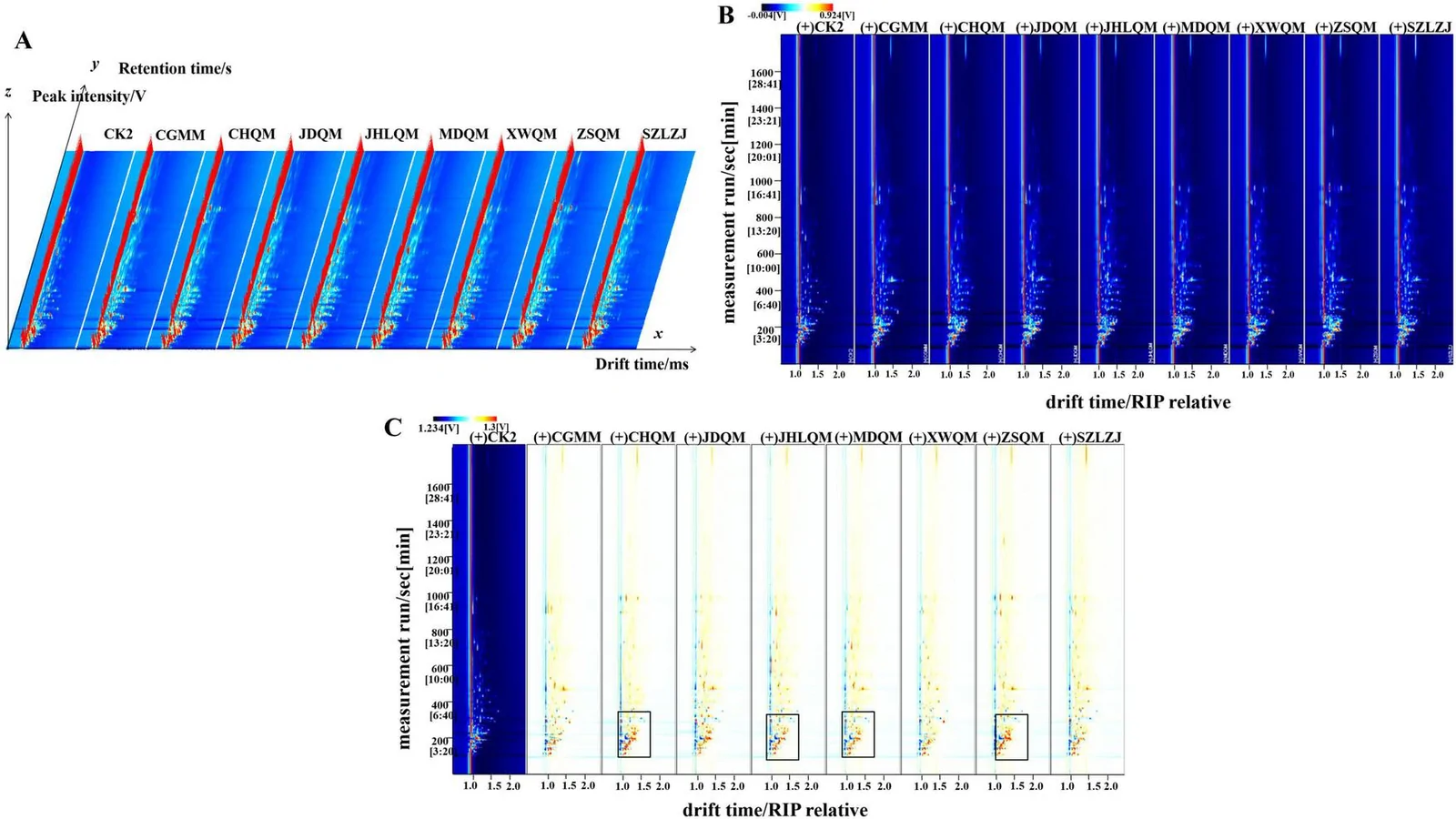
Gas chromatography-ion mobility spectrometry (GC-IMS) spectra of cigar tobacco leaves (CTLS) inoculated with different mold spore suspensions and incubated for 5 days. **(A)** 3D and **(B)** 2D topographic plots. **(C)** A difference plot constructed using the 2D GC-IMS spectrum of CK2 group as the reference.

Figures 3A, B display the 3D and 2D GC-IMS spectra of CTLs inoculated with different mold spore suspensions, respectively. The sample groups included the non-inoculated control (CK2) and CTLs inoculated with specific strains: *T. longibrachiatum* (CGMM), *P. chrysogenum* (CHQM), *A. sydowii* (JDQM), *A. pseudoglaucus* (JHLQM), *A. montevidensis* (MDQM), *A. chevalieri* (XWQM), *A. versicolor* (ZSQM), and *T. funiculosus* (SZLZJ). In these topographic plots, the x-axis represents the ion drift time, the y-axis represents the gas chromatographic retention, and the signal intensity (indicated by peak height in 3D or dot color in 2D) correlates positively with the VOC concentration. Both figures intuitively demonstrate substantial variations in VOC profiles among the sample groups.

To further highlight the dynamic changes in VOCs, a difference plot (Figure 3C) was constructed using the 2D spectrum of the CK2 group as the reference. In this plot, regions with a white background denote VOC concentrations identical to the reference, while red and blue regions indicate higher and lower concentrations, respectively. The CHQM, JHLQM, MDQM, and ZSQM groups exhibited significantly more intensive red regions (as highlighted by the black boxes in Figure 3C) compared to the other inoculated groups. These four samples perfectly correspond to the “high moldogenicity” group defined in Table 2. This finding strongly indicates that high-moldogenicity strains induced much more dramatic alterations in the volatile profiles of CTLs than low-moldogenicity strains.

#### 3.3.2 Characterization of VOCs

A total of 100 peaks were detected by GC-IMS across nine sample groups, with 66 VOCs being identified. These VOCs included 6 esters, 19 aldehydes, 11 alcohols, 15 ketones, 5 sulfur-containing compounds, 3 pyrazines, 2 furans, 2 aromatic compounds, 1 pyran, 1 acid, and 1 alkene (Table 3). These results indicated that aldehydes, alcohols, and ketones were the main VOCs in CTLs inoculated with different mold spore suspensions (Yu et al., 2023). Esters, produced by the esterification of carboxylic acids and alcohols (Sun et al., 2023), impart fruity flavors (Rubén et al., 2019). Hexanal contributes a grassy aroma, while heptanal imparts nutty and sweet apricot flavors (Ben et al., 2017). Meanwhile, 1-octen-3-ol contributes a mushroom-like aroma (Combet et al., 2006), and ketones often exhibit strong fruity and floral aromas (Zhou et al., 2022). For example, 2-propanone imparts apple and pear aromas, while 2-heptanone contributes a pear-like aroma. Pyrazines, intermediates in the Maillard reaction provide nutty, creamy, and baked aromas (Scalone et al., 2015), with 2-methylpyrazine producing a nutty aroma. Sulfides typically emit pungent aromas reminiscent of cooked vegetables, onions, and garlic (Lei et al., 2020).

**TABLE 3:** Total peak volumes of different categories of volatile organic compounds (VOCs) identified in cigar tobacco leaves (CTLs) inoculated with different mold spore suspensions.

Number	Compound	CAS#	RI	RT (sec)	Dt (a.u.)	Code*
Esters (6)						
1	Linalyl acetate	115-95-7	1557.8	1093.078	1.22	IMS9
2	1-Butanol, 3- methyl-, acetate-M	123-92-2	1129.9	343.427	1.31	IMS42
	1-Butanol, 3- methyl-, acetate-D		1129.6	343.040	1.75	IMS43
3	Amyl acetate	628-63-7	1180.8	411.795	1.31	IMS47
4	Ethyl pentanoate	539-82-2	1148.5	366.905	1.26	IMS66
5	Butanoic acid 3-methyl, ethyl ester-M	108-64-5	1075.6	285.667	1.26	IMS73
	Butanoic acid 3-methyl, ethyl ester-D		1076.5	286.416	1.65	IMS74
6	Propyl propanoate	106-36-5	1043.1	258.075	1.22	IMS89
Aldehydes (19)						
1	Alpha-Tolualdehyde	122-78-1	1623.2	1265.622	1.25	IMS1
2	Benzaldehyde-M	100-52-7	1501.5	963.672	1.16	IMS2
	Benzaldehyde-D		1500.3	961.102	1.47	IMS3
3	2-furaldehyde-M	98-01-1 98-01-1	1463.5	885.105	1.09	IMS5
	2-furaldehyde-D		1463.0	884.012	1.34	IMS15
4	2-Hydroxybenzaldehyde	90-02-8	1669.9	1405.449	1.15	IMS7
5	1-nonanal	124-19-6	1398.2	764.744	1.47	IMS12
6	(E)-2-hexenal-M	6728-26-3	1222.5	475.189	1.18	IMS23
	(E)-2-hexenal-D	6728-26-3	1225.9	480.67	1.51	IMS97
7	3-Methyl-2-butenal-M	107-86-8	1207.1	451.15	1.09	IMS26
	3-Methyl-2-butenal-D	107-86-8	1207.5	451.722	1.36	IMS27
8	Heptaldehyde-M	111-71-7	1190.2	425.966	1.33	IMS28
	Heptaldehyde-D	111-71-7	1190.2	425.934	1.70	IMS94
9	(E)-2-Pentenal-M	1576-87-0	1140.5	356.712	1.11	IMS33
	(E)-2-Pentenal-D	1576-87-0	1139.2	354.995	1.36	IMS34
10	1-hexanal-M	66-25-1	1096.5	304.902	1.27	IMS71
	1-hexanal-D	66-25-1	1096.0	304.402	1.57	IMS72
11	(E)-2-Butenal	123-73-9	1060.4	272.427	1.20	IMS75
12	n-Pentanal	110-62-3	998.8	224.766	1.43	IMS79
13	3-Methyl butanal	590-86-3	927.6	188.102	1.41	IMS85
14	Propanal	123-38-6	795.5	135.623	1.15	IMS88
15	Acrolein	107-02-8	862.0	159.919	1.06	IMS92
16	Octanal	124-13-0	1292.7	602.251	1.40	IMS39
17	(E)-2-Heptenal	18829-55-5	1333.4	661.402	1.26	IMS18
18	3-(methylsulfanyl)propanal	3268-49-3	1448.5	855.809	1.09	IMS14
19	(Z)-2-pentenal	1576-86-9	1108.9	318.642	1.10	IMS67
Alcohols (11)						
1	1-Octen-3-ol	3391-86-4	1456.4	871.068	1.17	IMS6
2	2-ethyl hexanol	104-76-7	1495.1	949.956	1.40	IMS8
3	2-butoxyethanol	111-76-2	1402.6	772.338	1.20	IMS10
4	1-Pentanol	71-41-0	1257.4	534.527	1.25	IMS21
5	2-methyl-1-butanol-M	137-32-6	1214.2	462.025	1.24	IMS24
	2-methyl-1-butanol-D	137-32-6	1213.5	460.88	1.47	IMS25
6	1-Butanol-M	71-36-3	1170.4	396.777	1.18	IMS30
	1-Butanol-D	71-36-3	1168.9	394.719	1.39	IMS65
7	1-Penten-3-ol	616-25-1	1165.4	389.76	0.95	IMS51
8	(Z)-2-pentenol	1576-95-0	1310.8	628.824	1.45	IMS61
9	1-Propanol, 2-methyl-M	78-83-1	1103.8	312.896	1.17	IMS68
	1-Propanol, 2-methyl-D	78-83-1	1105.8	315.144	1.36	IMS69
10	Ethanol	64-17-5	940.9	194.419	1.13	IMS91
11	2-Pentanol	6032-29-7	1127.2	340.134	1.21	IMS99
Ketones (15)						
1	1-(2-furanyl) ethanone	1192-62-7	1490.9	940.988	1.11	IMS13
2	6-Methyl-5-hepten-2-one	110-93-0	1347.1	682.066	1.18	IMS17
3	1-Hydroxy-2-propanone	116-09-6	1311.2	629.338	1.23	IMS19
4	2-Heptanone-M	110-43-0	1189.1	424.249	1.26	IMS29
	2-Heptanone-D	110-43-0	1186	419.505	1.62	IMS57
5	Cyclohexanone	108-94-1	1289.8	596.38	1.15	IMS52
6	Cyclopentanone	120-92-3	1193.1	430.221	1.11	IMS54
7	3-Octanone-M	106-68-3	1262.6	544.015	1.30	IMS93
	3-Octanone-D	106-68-3	1259.7	538.712	1.72	IMS59
8	4-Heptanone	123-19-3	1132.1	346.164	1.59	IMS62
9	1-Penten-3-one	1629-58-9	1037.3	253.441	1.31	IMS77
10	4-Methyl-2-pentanone	108-10-1	1022.4	241.95	1.48	IMS78
11	3-Pentanone	96-22-0	996.9	223.424	1.36	IMS80
12	2-Butanone	78-93-3	916.9	183.17	1.23	IMS82
13	2-propanone	67-64-1	831.5	148.257	1.12	IMS87
14	2-Hexanone	591-78-6	1092.2	300.871	1.50	IMS100
15	2-Octanone	111-13-7	1292.7	602.272	1.33	IMS95
Sulfur-containing compounds (5)						
1	2,5-Dimethyl thiophene	638-02-8	1159.6	381.804	1.08	IMS45
2	Diethyl disulfide	110-81-6	1222.3	474.9	1.14	IMS58
3	2-Methyl-3-furanthiol	28588-74-1	1304.1	619.525	1.15	IMS63
4	Dimethyl trisulfide	3658-80-8	1373.5	723.502	1.31	IMS64
5	3-(methylthio)-1-propene	10152-76-8	971.5	209.718	1.03	IMS98
Pyrazines (3)						
1	2-Methylpyrazine	109-08-0	1269.9	557.607	1.09	IMS36
2	2-Ethyl-5-methylpyrazine	13360-64-0	1387.3	746.313	1.17	IMS50
3	Pyrazine	290-37-9	1223.2	476.253	1.05	IMS60
Furans (2)						
1	2-pentyl furan	3777-69-3	1234.2	494.342	1.25	IMS46
2	Tetrahydrofuran	109-99-9	884.7	169.151	1.23	IMS86
Aromatics (2)						
1	Ethyl benzene	100-41-4	1135.6	350.416	1.07	IMS35
2	Ethenyl benzene	100-42-5	1262.5	543.817	1.44	IMS96
Pyrans (1)						
1	Rose oxide	16409-43-1	1352.3	690.088	1.35	IMS56
Acids (1)						
1	Acetic acid	64-19-7	1462.2	882.553	1.05	IMS4
Alkenes (1)						
1	Delta-cadinene	483-76-1	1764.9	1738.608	1.46	IMS11

*Each code is a randomly generated code for a total of 100 peaks.

**FIGURE 4:**
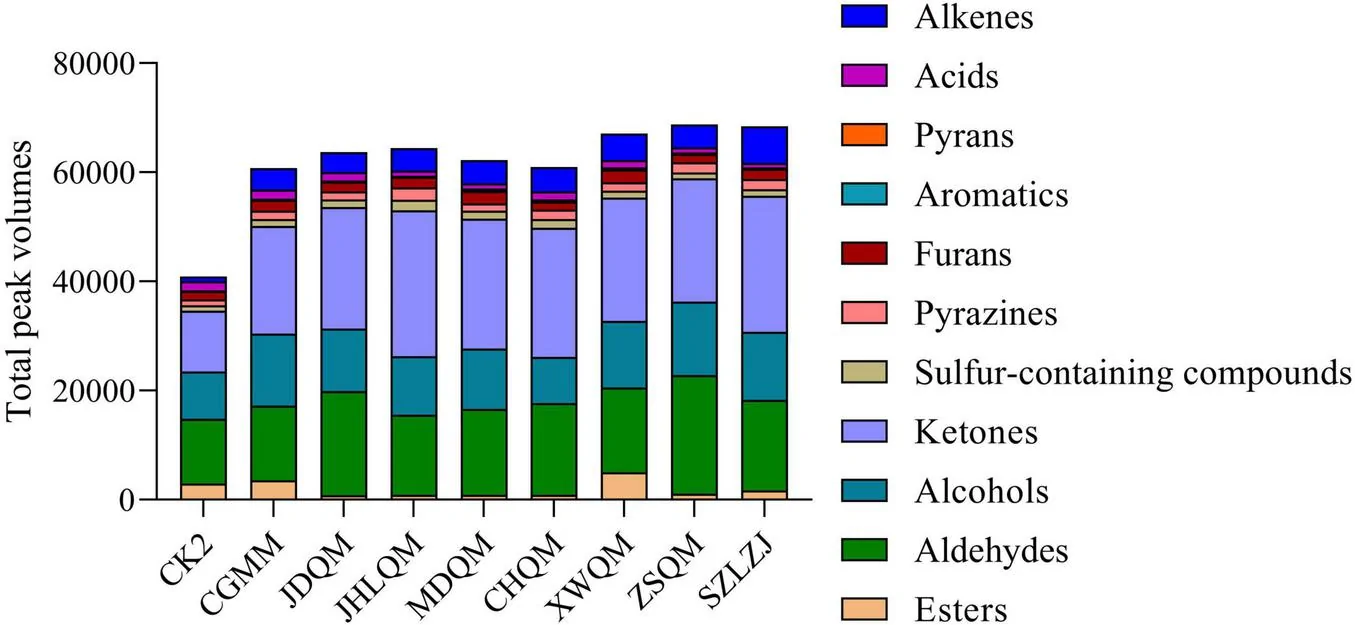
Peak volumes for different categories of volatile organic compounds (VOCs) identified in cigar tobacco leaves (CTLs) inoculated with different mold spore suspensions .

The identified VOCs were analyzed according to their categories, and the comparative results are shown in Figure 4. It should be noted that due to concentration-dependent ionization in GC-IMS, certain compounds were detected in both monomeric (M) and dimeric (D) forms. When calculating the total number of identified VOCs and the percentage distribution of chemical categories, the M and D forms of the same chemical were unified and counted as a single unique compound. Given the semi-quantitative nature of GC-IMS, total peak volumes were used to represent the relative abundance of these chemical classes. Compared to the ester abundance in control (CK2) CTLs, those in CTLs inoculated with the spore suspensions of *T. longibrachiatum* and *A. chevalieri* (the CGMM and XWQM groups, respectively) showed an increasing trend. In contrast, the ester abundances in CTLs inoculated with the other six mold species showed a decreasing trend. The relative abundance of aldehydes, ketones, and alkenes increased significantly after CTL inoculation with any of the mold spore suspension. Compared to the alcohol abundance in CK2, that in CTLs inoculated with *P. chrysogenum* showed a minimal change, whereas inoculation with the other seven species resulted in an increasing trend. The abundance of sulfur-containing compounds, pyrazines, and furans exhibited a slightly increasing trend following inoculation with any mold species. Compared to the pyranoid abundance in CK2, those in CTLs inoculated with *A. montevidensis*, *P. chrysogenum*, and *A. versicolor* increased, whereas those in the other five groups remained relatively unchanged. Notably, these three species were not only associated with increased pyrans level but also induced a more severe moldy appearance in CTLs, suggesting a potential association between pyran accumulation and severe mold manifestation, though its definitive role as a universal mold marker requires further investigation. Finally, the acid abundance in CTLs inoculated with any mold species showed a general decreasing trend.

#### 3.3.3 Semi-quantification and cluster analysis of VOCs

**FIGURE 5:**
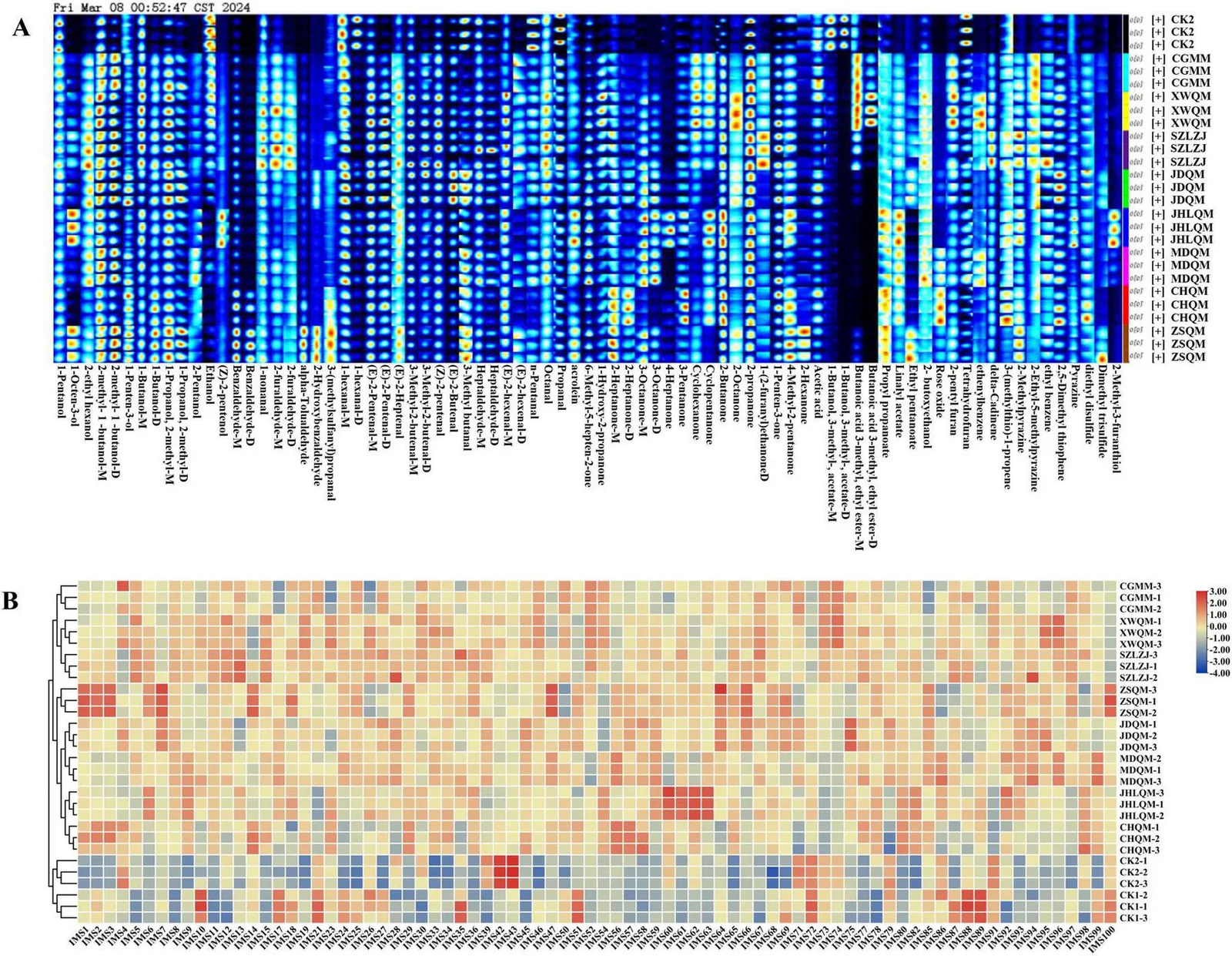
**(A)** Volatile organic compounds (VOC) fingerprints of cigar tobacco leaves (CTLs) inoculated with different mold spore suspensions. **(B)** A heatmap showing differences in VOC composition among different groups.

Figure 5 shows the VOC fingerprints of CTLs inoculated with different mold spore suspensions. The color intensity represents the relative abundance of a VOC in a sample group: the redder the color, the higher the relative abundance of the VOC in that group. Significant differences in VOC composition were observed between the CK2 and CTLs inoculated with different mold spore suspensions (Figure 5A). The relative abundances of the following VOCs increased after CTL inoculation with different mold spore suspensions: 1-octen-3-ol, 2-ethylhexanol, butanol, 2-methylpropanol, furfural, benzaldehyde, hexenal, 3-methyl-2-butenal, 2-heptanone, 3-octanone, 3-pentanone, 2-butanone, 2-pentylfuran, 5-dimethylthiophene, and 2-methylpyrazine. Conversely, the relative abundances of the following VOCs decreased after CTL inoculation: 3-methylbutyl acetate, propionaldehyde, valeraldehyde, hexanal, ethanol, and pentanol.

Furthermore, specific VOC patterns were observed for different mold strains. The relative abundances of cyclohexanone; butanoic acid, 3- methyl-, ethyl ester (M); and 2-pentyl furan increased after CTL inoculation with *T. longibrachiatum* spore suspension. The relative abundances of butanoic acid, 3- methyl-, ethyl ester (M and D); 2-furaldehyde (M and D); 1-nonanal; 1-hydroxy-2-propanone; cyclohexanone; 2-octanone; 2-pentyl furan; and ethenylbenzene increased after CTL inoculation with *A. chevalieri* spore suspension. The relative abundances of 1-nonanal, 2-furaldehyde (M and D), cyclohexanone, 1-(2-furanyl) ethanone, and delta-cadinene increased after CTL inoculation with *T. funiculosus* spore suspension. The relative abundances of (E)-2-butenal, 1-propanol, and 2-methyl-1-butanol (D) increased after CTL inoculation with *A. sydowii* spore suspension. The relative abundances of linalyl acetate, propyl propanoate, acrolein, 1-octen-3-ol, (Z)-2-pentenol, 4-heptanone, 2-methyl-3-furanthiol, and pyrazine increased after CTL inoculation with *A. pseudoglaucus* spore suspension. The relative abundances of 2-pentanol, rose oxide, propyl propanoate, linalyl acetate, and ethenylbenzene increased after CTL inoculation with *A. montevidensis* spore suspension. After CTL inoculation with *P. chrysogenum* spore suspension, the relative abundances of rose oxide, propyl propanoate, linalyl acetate, benzaldehyde (M and D), 3-(methylsulfanyl) propanal, 2-heptanone (M and D), and 3-pentanone increased, whereas the relative abundances of ethanol decreased. Finally, the relative abundances of acrolein, 1-octen-3-ol, 1-propanol, 2-methyl (D form), benzaldehyde (M and D), 2-heptanone (M), alpha-tolualdehyde, 3-(methylsulfanyl) propanal, 2-hexanone, propyl propanoate, and ethyl pentanoate increased after CTL inoculation with *A. versicolor* spore suspension.

A heatmap visually represents data variations through color intensity and facilitates the hierarchical clustering of samples with high correlated VOC profiles. In this study, the 66 VOC peaks detected across the sample groups (CK1, CK2, XWQM, SZLZJ, ZSQM, CHQM, JHLQM, JDQM, and MDQM) were subjected to hierarchical cluster analysis using TBtools software (v2.0). As shown in Figure 5B, the sample groups were categorized into three major clusters: the CK1 and CK2 formed the first cluster; the CGMM, XWQM, and SZLZJ groups formed the second cluster, and the ZSQM, CHQM, JHLQM, JDQM, and MDQM groups formed the third cluster. To further identify mold-associated marker VOCs, partial least squares-discriminant analysis (PLS-DA) was subsequently conducted on the VOCs detected across these groups.

#### 3.3.4 Chemometric analysis of VOCs

**FIGURE 6:**
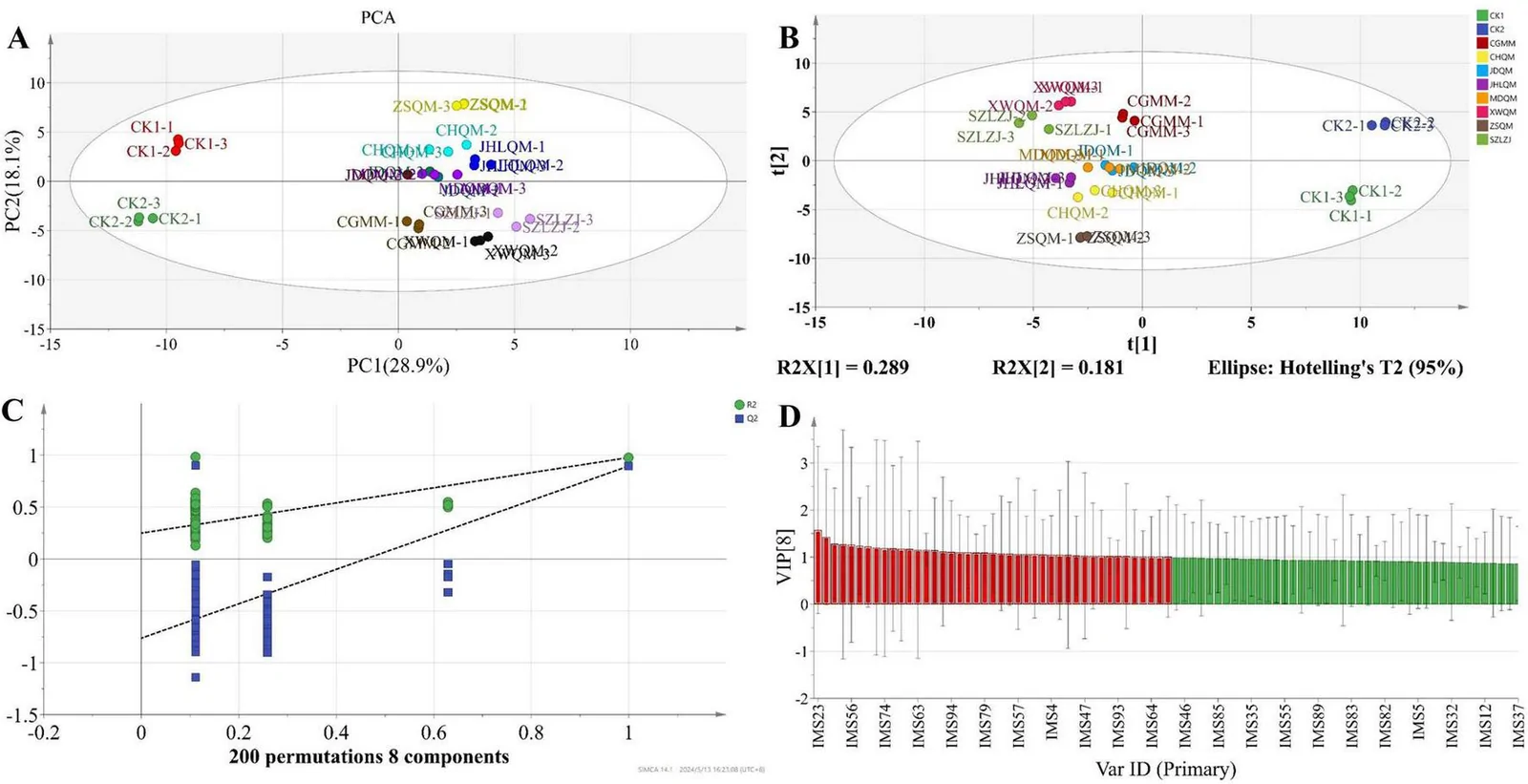
(A) Principal component analysis (PCA) and (B) partial least squares discriminant analysis (PLS-DA) plots showing the separation of cigar tobacco leaves (CTLs) inoculated with different mold spore suspensions based on their VOC profiles. (C) Permutation test results validating the PLS-DA model. (D) A variable importance in projection (VIP) diagram highlighting the differential VOCs identified by the PLS-DA model.

To explore the difference in VOC profiles among CTLs inoculated with different mold spore suspensions, an unsupervised Principal Component Analysis (PCA) was first performed. The data matrix consisted of the normalized relative peak volumes of the 66 identified VOCs across all sample groups. Data were mean-centered and scaled to unit variance (UV) prior to analysis. As an unsupervised method, PCA does not assign class labels to samples. The PCA score plot (Figure 6A) showed that the variance contributions of the first (PC1) and second (PC2) principal components were 28.8% and 18.1%, respectively (cumulative contribution of 47%). The CK1 and CK2 were clearly separated from the mold-inoculated samples groups. Furthermore, different mold-inoculated samples clustered in distinct quadrants, reflecting significant inherent VOC differences driven by the specific mold strains.

To further identify the differential VOCs responsible for distinguishing these groups, a supervised Partial Least Squares-Discriminant Analysis (PLS-DA) was performed. The class variable was defined by the specific mold inoculation treatment. The PLS-DA score plot (Figure 6B) confirmed clear separation among the groups without any strong outliers, as all data points fell within in the 95% confidence ellipse (Hotelling’s T2 interval) (Zhao G. et al., 2024). The model metrics were evaluated using 200 permutations through cross-validated analysis of variance (Figure 6C). The fit index of the independent variable (R^2^X) was 0.912, the fit index of the dependent variable (R^2^Y) was 0.871, the model prediction index (Q^2^) was 0.541, and the intersection of the Q^2^ regression line with the vertical axis was less than 0. These results indicated that the model did not overfit, confirming its validity and highly reliable for differentiating molds-induced VOC profiles.

Additionally, the spatial distribution of the samples in both PCA and PLS-DA score plots exhibited a strong correlation with their moldogenicity classifications. The four high-moldogenicity strain (*A. montevidensis*, *P. chrysogenum*, *A. pseudoglaucus*, and *A. versicolor*) and the three low-moldogenicity strains (*T. funiculosus*, *T. longibrachiatum*, and *A. chevalieri*) were distinctly separated into two main clusters along the principal components. This distinct separation indicates that high and low moldogenicity strains drive fundamentally different trajectories of VOC alteration in tobacco leaves. Interestingly, *A. sydowii –*which exhibited low colony counts and visual scores – was positioned much closer to the high-moldogenicity cluster in the chemometric space. This phenomenon suggests that while its physical colonization rate may be relatively moderate, its secondary metabolism and capacity to alter the tobacco volatilome are highly active, further highlighting the sensitivity of GC-IMS in revealing the underlying metabolic shifts during mold contamination.

To identify the differential VOCs driving this separation, the contribution of each variable to the PLS-DA model was assessed by calculating the variable importance in projection (VIP) value, with a larger VIP value indicating a more significant difference (Song et al., 2021). Based on the screening criterion of VIP > 1, a total of 39 differential VOCs were identified across the sample groups (Figure 6D). They included 11 aldehydes, 9 ketones, 6 alcohols, 4 esters, 2 pyrazines, 2 sulfur-containing compounds, 2 furans, 1 pyran, 1 aromatic compound, and 1 acid.

#### 3.3.5 Analysis of mold marker compounds

**FIGURE 7:**
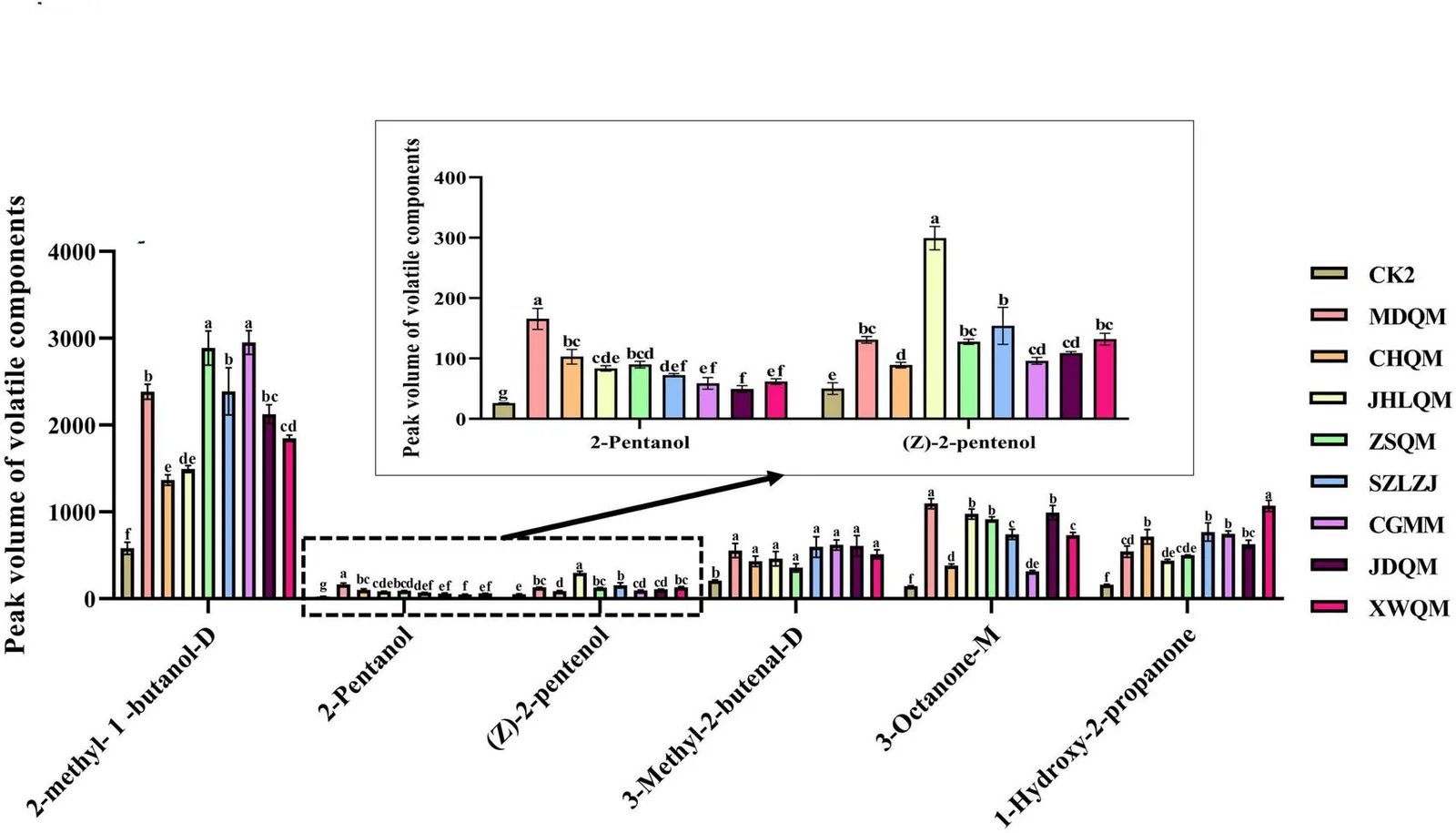
Relative abundance and comparison of six broad-spectrum volatile marker compounds in cigar tobacco leaves inoculated with different molds. Different lowercase letters indicate statistically significant differences among the sample groups (*P* < 0.05) as determined by one-way ANOVA followed by Tukey’s HSD test.

To screen for specific volatile markers, the 39 differential VOCs (VIP > 1) identified by the PLS-DA model were subjected to one-way ANOVA to evaluate their significant differences among the sample groups (Supplementary Table 1). To develop a robust and broad-spectrum early warning strategy for mold contamination in cigar tobacco leaves (CTLs), we further screened the differential VOCs based on their universality across different fungal infections. By comparing the volatilome profiles, six VOCs—2-pentanol, 2-methyl-1-butanol (D), (Z)-2-pentenol, 3-methyl-2-butenal (D), 3-octanone (M), and 1-hydroxy-2-propanone—were identified as potential broad-spectrum markers (Figure 7). As shown in Supplementary Table 1, the relative abundances of these six compounds in all eight mold-inoculated groups were significantly higher than those in the CK2 (*P* < 0.05). This universal upregulation, regardless of the specific infecting strain, demonstrates their excellent broad-spectrum applicability for the general early detection of mold outbreaks.

Furthermore, quantitative analysis revealed a compelling trend regarding 2-pentanol as an indicator of infection severity. The relative abundance of 2-pentanol was prominently higher in CTLs inoculated with high-moldogenicity strains (e.g., MDQM, CHQM, JHLQM, and ZSQM) compared to those infected by low-moldogenicity strains. Additionally, typical fungal volatile organic compounds (TFVOCs) such as 2-pentanol and 3-octanone are closely associated with fungal growth metabolism (Matysik et al., 2008). Therefore, this panel of six VOCs can serve as universal chemical indicators for mold contamination, with 2-pentanol providing additional diagnostic value for assessing the severity and high-risk nature of the fungal infection during CTL aging.

## 4 Discussion and future perspectives

### 4.1 Identification and biological mechanisms of core early warning mold markers

In this study, through chemometric screening (VIP > 1 and *P* < 0.05), six VOCs were initially identified as broad-spectrum markers. Among them, three key compounds—3-octanone, 2-pentanol, and 2-methyl-1-butanol—exhibited the most prominent and consistent responses across all dominant molds and were thus selected as the core early-warning markers of CTLs mold contamination.

The identification of these specific markers aligns perfectly with classical fungal volatilomics. 3-octanone (M) is widely recognized as a typical fungal secondary metabolite, formed via the enzymatic oxidation of lipids (Combet et al., 2006). Its presence serves as a direct indicator of fungal colonization and active lipid degradation in the tobacco matrix (Kaminski et al., 1974). 2-pentanol has been previously highlighted by Matysik et al. (2008) alongside 3-octanone and 1-octen-3-ol as critical volatile indicator of microbial activity. In our study, 2-pentanol exhibited exceptionally high relative abundances, particularly in the high-moldogenicity strains (e.g., MDQM, CHQM, JHLQM, and ZSQM), suggesting its strong potential not only as a broad-spectrum marker but also as a quantitative indicator of mold severity. Furthermore, 2-methyl-1-butanol is a well-documented product of amino acid (isoleucine) degradation via the Ehrlich pathway (Yogiswara and Verstrepen, 2026).

Fundamentally, the observed variations in VOCs among different strains can be intrinsically attributed to the biological basis of their moldogenicity potential. Highly-moldogenicity strains (such as *A*. *montevidenis* and *P*. *chrysogenum*) likely possess more aggressive enzymatic arsenals—including lipases, proteases, and cellulases—which enable them to rapidly degrade the tobacco matrix. This intense metabolic activity and severe host tissue destruction result in a higher accumulation and higher concentration of specific stress and decay-related VOCs compared to low-moldogenicity strains.

### 4.2 Limitations and future perspectives

Despite the promising findings regarding VOC-based early warning, several limitations of this study must be acknowledged to guide future research. First, regarding the sampling strategy, the primary objective of this study was to successfully isolate and characterize the dominant spoilage mold strains responsible for severe contamination during specific typical processing stages, rather than to conduct an exhaustive national geographical survey. Future studies should involve large-scale, multi-region sampling to further validate and expand our findings. Second, while this study established a quantitative classification of moldogenicity based no visual scores and mold counts, a comprehensive evaluation should ideally incorporate additional factors such as mycotoxin production potential, dynamic spoilage rates, and direct sensory influence. Future studies should integrate these dimensions with VOCs profiled to further refine this concept.

Furthermore, although GC-IMS demonstrated great efficacy for VOC-based early warning, product safety cannot be fully guaranteed by VOC profiles and fungal growth alone. Genera such as *Aspergillus* and *Penicillium* contain potentially toxigenic strains; therefore, future research must integrate LC-MS/MS (Liquid Chromatography-Tandem Mass Spectrometry) techniques to screen mycotoxin biomarkers and assess toxin biosynthesis-related genes to provide a comprehensive safety evaluation. Finally, although this cross-sectional study successfully identified strain-specific markers at a single point (day 5), fungal infection is a highly dynamic process. Future studies must incorporate time-series dynamic monitoring for highly dynamic process (e.g., tracking VOC profiles continuously from day 1 to day 7) to fully validate the diagnostic performance of these markers for early-stage infection.

## 5 Conclusion

In this study, eight dominant mold strains isolated from moldy cigar tobacco leaves (CTLs) were identified and quantitatively classified into high-moldogenicity (*A. montevidensis*, *P. chrysogenum*, *A. pseudoglaucus*, and *A. versicolor*) and low-moldogenicity strains (*T. funiculosus*, *T. longibrachiatum*, *A. chevalieri*, and *A. sydowii*) based on a combined visual scoring and colony counting evaluation system. Using GC-IMS, the VOCs profiles of CTLs inoculated with these distinct strains were successfully characterized. A total of 66 VOCs were identified. Crucially, multivariate chemometric analyses (PCA and PLS-DA) not only clearly differentiated mold-contaminated samples from control groups but also clustered the strains according to their defined mold-inducing potential. Through strict variable screening (VIP > 1, *P* < 0.05), three VOCs—3-octanone (M), 2-pentanol, and 2-methyl-1-butanol—were identified as robust, broad-spectrum early-warning markers for mold contamination. Additionally, 2-pentanol exhibited a distinctly higher abundance in high-moldogenicith strains, highlighting its potential as a quantitative indicator of infection severity. Overall, this study demonstrates that GC-IMS is a rapid and highly effective analytical tool for profiling dynamic VOC changes during fungal infection. These findings provide valuable insights into the biological mechanisms of moldogenicity and offer a promising VOC-based targeted monitoring strategy to ensure safe cigar tobacco production and prevent early-stages mold outbreaks.

## Data Availability

The original contributions presented in this study are included in this article/Supplementary material, further inquiries can be directed to the corresponding authors.
