# Correction: Moldogenicity of dominant culturable molds in cigar tobacco leaves and GC-IMS–based analysis of volatile organic compounds

**DOI:** 10.3389/fmicb.2026.2004227

**Published:** 2026-09-28

**Authors:** Kuo Huang, Ge Zhang, Mengmeng Liu, Shaoxin Yang, Chen Chen, Beibei Zhu, Min Wei, Yong Pan, Xiaoyan Gao, Dong Li, Delong Xu, Fang Zheng, Fang Xue, Changwen Ye

**Affiliations:** 1 Zhengzhou Tobacco Research Institute, China National Tobacco Corporation, Zhengzhou, China; 2 College of Food Science and Technology, Huazhong Agricultural University, Wuhan, China; 3 College of Plant Protection, Southwest University, Chongqing, China; 4 China Tobacco Shandong Industrial Co., Ltd., Jinan, China; 5 Cigar Technology Innovation Center of China Tobacco, China Tobacco Sichuan Industrial Co., Ltd., Chengdu, China; 6 Technology Centre of Hubei China Tobacco Industry Co., Ltd., Wuhan, China

**Keywords:** cigar tobacco leaves, GC-IMS, moldogenicity, molds, VOCs

An incorrect grant number was provided for Major Science and Technology Program of China National Tobacco Corporation. The correct number is 110202201034(XJ-05).

The original version of this article has been updated.

