# Supplementary material for: Moldogenicity of dominant culturable molds in cigar tobacco leaves and GC-IMS–based analysis of volatile organic compounds
Source: Front Microbiol. 2026 Aug 28;17:1843382. doi: 10.3389/fmicb.2026.1843382 (PMC13562250; doi:10.3389/fmicb.2026.1843382)
Supplement: Supplementary file 1 [file Supplementary_file_1.docx]

Supplementary Material

**Supplementary Fig. S1.** The results of MALDI-TOF MS of strains.

## Supplementary Table S1. The results of significant differences among these groups.

| Compound Name | Compound number | CK1 | CK2 | CGMM | CHQM | JDQM | JHLQM | MDQM | XWQM | ZSQM | SZLZJ |
| --- | --- | --- | --- | --- | --- | --- | --- | --- | --- | --- | --- |
| (E)-2-hexenal-M | IMS23 | 473.00±52.35^bcd^ | 418.83±32.76^cd^ | 289.03±24.56^e^ | 594.00±28.43^ab^ | 361.08±56.02^de^ | 580.20±40.19^ab^ | 444.47±35.73^cd^ | 637.31±43.37^a^ | 449.57±20.67^cd^ | 529.68±66.76^abc^ |
| 2-Octanone | IMS95 | 44.72±5.46^de^ | 34.87±2.79^e^ | 46.94±6.44^cde^ | 50.72±4.19^cde^ | 103.56±17.02^a^ | 58.63±4.22^bcd^ | 70.33±6.43^bc^ | 115.18±8.81^a^ | 78.96±3.98^b^ | 64.61±7.81^bcd^ |
| 3-Methyl-2-butenal-M | IMS26 | 734.69±130.98^a^ | 640.61±7.05^ab^ | 454.04±40.96^c^ | 569.01±55.38^abc^ | 658.40±70.28^ab^ | 695.56±56.41^a^ | 672.92±32.82^ab^ | 766.99±67.68^a^ | 491.96±22.40^bc^ | 731.56±44.96^a^ |
| (E)-2-Butenal | IMS75 | 765.31±228.12^c^ | 842.12±127.68^c^ | 1693.65±449.17^b^ | 1194.76±126.11^bc^ | 3816.07±112.42^a^ | 1771.96±127.78^b^ | 1552.16±297.38^b^ | 1662.62±103.05^b^ | 907.08±66.01^b^ | 1716.92±89.86^b^ |
| Rose oxide | IMS56 | 18.76±5.31^e^ | 18.52±3.49^e^ | 29.81±3.54^e^ | 259.16±3.91^a^ | 68.75±3.91^d^ | 31.12±2.04^e^ | 211.26±10.41^b^ | 21.84±4.22^e^ | 105.14±1.29^c^ | 22.87±2.85^e^ |
| ethenylbenzene | IMS96 | 41.21±1.72^d^ | 37.02±5.73^d^ | 43.98±1.49^d^ | 53.62±5.57^d^ | 59.06±11.10^d^ | 67.70±6.08^d^ | 190.06±8.97^b^ | 247.80±36.67^a^ | 73.51±14.15^d^ | 121.03±13.52^c^ |
| 2-Pentanol | IMS99 | 111.71±16.23^b^ | 26.18±0.77^g^ | 58.91±9.73^ef^ | 103.08±12.13^bc^ | 49.60±5.46^f^ | 83.65±4.39^cde^ | 165.80±17.53^a^ | 61.93±4.65^ef^ | 90.03±5.46^bcd^ | 72.51±2.50^def^ |
| Butanoic acid 3-methyl, ethyl ester-D | IMS74 | 59.15±10.54^e^ | 317.84±41.19^c^ | 1510.81±20.60^b^ | 43.04±2.99^e^ | 35.13±4.14^e^ | 30.53±3.32^e^ | 29.61±3.44^e^ | 2633.03±61.17^a^ | 49.89±2.19^e^ | 228.35±18.69^d^ |
| 4-Heptanone | IMS62 | 29.11±1.28^e^ | 63.51±5.44^cd^ | 50.24±7.35^de^ | 102.92±12.61^b^ | 82.59±5.72^bc^ | 317.74±20.79^a^ | 32.96±4.74^e^ | 37.38±3.87^de^ | 88.63±9.33^b^ | 44.89±3.06^de^ |
| 1-Octen-3-ol | IMS6 | 82.61±14.01^f^ | 69.54±13.16^f^ | 283.22±61.68^de^ | 131.73±31.86^ef^ | 365.26±31.46^d^ | 1533.28±15.63^a^ | 345.58±24.68^d^ | 465.48±136.50^d^ | 1345.89±115.75^b^ | 780.78±102.59^c^ |
| 2-Ethyl-5-methylpyrazine | IMS50 | 113.93±8.21^b^ | 32.51±7.11^c^ | 180.84±7.33^a^ | 109.12±33.20^b^ | 142.14±12.17^ab^ | 104.26±16.21^b^ | 116.40±20.38^b^ | 149.18±22.66^ab^ | 36.00±5.32^c^ | 116.09±24.71^b^ |
| 2-Methyl-3-furanthiol | IMS63 | 29.64±2.32^f^ | 39.27±5.46^f^ | 142.82±12.70^bc^ | 63.33±10.40^ef^ | 84.50±2.86^de^ | 642.15±16.91^a^ | 174.97±30.30^b^ | 112.20±19.54^cd^ | 113.60±6.95^cd^ | 116.18±27.60^cd^ |
| 2-methyl- 1 -butanol-D | IMS25 | 2429.45±374.01^b^ | 581.43±69.84^f^ | 2951.27±136.51^a^ | 1368.41±59.09^e^ | 2126.62±107.22^bc^ | 1494.16±40.23^de^ | 2384.48±85.87^b^ | 1846.88±36.59^cd^ | 2888.04±196.97^a^ | 2389.99±271.35^b^ |
| Pyrazine | IMS60 | 612.55±14.13^d^ | 794.49±24.97^bc^ | 628.35±111.85^cd^ | 817.21±43.78^b^ | 715.48±7.74^bcd^ | 1152.63±86.51^a^ | 573.42±20.02^d^ | 687.49±20.46^bcd^ | 784.88±23.23^bc^ | 737.07±63.66^bcd^ |
| 1-(2-furanyl)ethanone | IMS13 | 102.25±1.77^cd^ | 72.62±11.29^d^ | 165.32±16.68^b^ | 116.64±11.37^c^ | 65.73±7.99^d^ | 166.49±12.80^b^ | 113.12±6.59^c^ | 164.11±14.97^b^ | 123.94±8.51^c^ | 273.79±30.03^a^ |
| Heptaldehyde-D | IMS94 | 104.67±18.18^c^ | 135.90±25.02^bc^ | 156.03±8.56a^bc^ | 140.98±11.43^abc^ | 242.44±18.56^ab^ | 153.62±25.90^abc^ | 269.46±40.67^a^ | 170.30±20.99^abc^ | 171.58±26.53^abc^ | 259.73±99.01^ab^ |
| 1-Propanol, 2-methyl-D | IMS69 | 814.00±219.90^abc^ | 275.35±70.01^d^ | 787.23±211.22^abc^ | 575.38±17.53^cd^ | 965.37±79.82^ab^ | 646.00±56.81^bcd^ | 456.03±11.66^cd^ | 617.92±25.66^bcd^ | 1102.79±164.13^a^ | 572.64±83.69^cd^ |
| 6-Methyl-5-hepten-2-one | IMS17 | 7024.65±166.70^a^ | 2051.73±521.77^de^ | 1524.70±89.48^e^ | 2565.52±494.88^cd^ | 4298.47±166.84^b^ | 4366.02±341.96^b^ | 4926.30±610.93^b^ | 3391.13±281.55^c^ | 3288.60±355.36^c^ | 3132.31±179.99^c^ |
| 3-Octanone-D | IMS59 | 47.53±12.47^c^ | 43.07±4.67^c^ | 71.43±7.16^c^ | 64.22±6.89^c^ | 300.90±11.59^a^ | 337.96±52.07^a^ | 359.45±55.25^a^ | 176.89±19.43^b^ | 226.97±32.27^b^ | 197.58±35.04^b^ |
| n-Pentanal | IMS79 | 1065.23±178.33^ab^ | 1229.17±121.73^a^ | 728.51±70.64^bcd^ | 432.05±84.68^d^ | 1210.11±153.45^a^ | 660.85±51.89^cd^ | 1077.02±137.56^ab^ | 1039.35±64.66^ab^ | 938.34±39.75^abc^ | 824.45±137.80^bc^ |
| 2,5-Dimethyl thiophene | IMS45 | 76.36±6.55^e^ | 139.35±5.21^de^ | 277.91±23.06^bc^ | 376.32±104.62^ab^ | 470.65±39.60^a^ | 272.22±16.58^bc^ | 372.88±17.08^ab^ | 365.04±30.30^ab^ | 221.97±21.99^cd^ | 261.58±22.72^bc^ |
| 2- butoxyethanol | IMS10 | 398.73±65.03^a^ | 151.30±28.66^c^ | 151.41±9.05^c^ | 148.20±11.08^c^ | 179.99±24.13^bc^ | 208.37±25.78^bc^ | 236.50±34.00^bc^ | 251.77±10.82^b^ | 163.62±17.04^bc^ | 224.21±17.10^bc^ |
| 2-Heptanone-D | IMS57 | 61.90±16.03^g^ | 74.41±16.60^g^ | 103.84±5.96^fg^ | 372.59±15.04^a^ | 252.51±7.05^b^ | 138.61±15.43^ef^ | 188.12±36.51^cd^ | 156.04±11.96^de^ | 224.68±6.61^bc^ | 165.08±17.56^de^ |
| Octanal | IMS39 | 93.69±10.13^e^ | 243.10±30.08^a^ | 171.95±22.11^bcd^ | 156.07±15.00^d^ | 224.17±5.50^a^ | 134.26±13.52^d^ | 213.43±14.37^ab^ | 206.02±18.63^abc^ | 164.31±6.71^cd^ | 228.30±18.29^a^ |
| Ethyl pentanoate | IMS66 | 39.34±4.29^d^ | 30.24±3.23^d^ | 47.57±9.26^cd^ | 36.32±0.98^d^ | 122.70±6.97^b^ | 27.88±2.35^d^ | 37.85±4.73^d^ | 69.64±8.49^c^ | 175.50±8.18^a^ | 72.45±23.57^c^ |
| Heptaldehyde-M | IMS28 | 520.92±61.69^b^ | 732.27±73.70 ^ab^ | 696.69±26.51^ab^ | 549.64±15.77^b^ | 850.23±20.31^a^ | 678.56±60.71^ab^ | 829.85±48.68^a^ | 737.39±75.89^ab^ | 675.42±36.04^ab^ | 884.09±166.37^a^ |
| Acetic acid | IMS4 | 906.73±71.69^a^ | 1696.68±378.17^a^ | 1678.23±721.63^a^ | 1490.81±268.71^a^ | 1570.25±184.41^a^ | 927.85±110.88^a^ | 883.83±46.06^a^ | 1351.18±498.97^a^ | 947.19±138.84^a^ | 758.58±42.89^a^ |
| Tetrahydrofuran | IMS86 | 1302.75±318.10^abc^ | 1352.03±46.72^ab^ | 1028.73±132.56^bcd^ | 871.96±79.40^cd^ | 981.39±35.85^bcd^ | 1377.28±31.81^ab^ | 1578.39±134.01^a^ | 1173.85±66.31^abcd^ | 771.49±58.39^d^ | 1144.01±209.85^abcd^ |
| ( Z)-2-pentenol | IMS61 | 45.40±8.48^e^ | 50.11±9.65^e^ | 96.20±5.62^cd^ | 89.17±4.67^d^ | 108.92±2.44^cd^ | 299.49±19.34^a^ | 131.01±5.56^bc^ | 132.00±9.98^bc^ | 127.95±4.38^bc^ | 154.09±30.60^b^ |
| 3-Methyl-2-butenal-D | IMS27 | 584.07±76.09^a^ | 209.61±9.00^b^ | 620.00±58.81^a^ | 432.83±60.14^a^ | 607.84±118.99^a^ | 461.17±84.08^a^ | 556.46±82.75^a^ | 512.75±52.33^a^ | 357.78±48.36^a^ | 596.69±117.24^a^ |
| Amyl acetate | IMS47 | 12.75±1.60^de^ | 10.03±0.91^f^ | 11.90±1.79^de^ | 21.04±0.27^c^ | 15.63±2.63^d^ | 13.89±0.72^de^ | 29.03±1.21^b^ | 9.69±1.19^f^ | 50.99±3.30^a^ | 16.98±1.18^d^ |
| 2-Hydroxybenzaldehyde | IMS7 | 142.26±28.15^c^ | 122.63±2.16^c^ | 137.24±19.08^c^ | 162.89±10.18^c^ | 414.12±11.29^b^ | 142.35±2.66^c^ | 171.95±16.25^c^ | 131.58±11.71^c^ | 636.28±67.26^a^ | 138.38±23.16^c^ |
| Cyclohexanone | IMS52 | 82.05±2.53^e^ | 115.06±8.46^de^ | 365.73±10.58^a^ | 148.97±10.18^cd^ | 158.79±6.11^cd^ | 167.39±5.70^cd^ | 80.23±10.45^e^ | 368.16±10.42^a^ | 203.18±31.70^c^ | 277.76±60.65^b^ |
| 1-hexanal-M | IMS71 | 821.09±57.17^cd^ | 1167.58±50.40^a^ | 1088.56±79.16^ab^ | 644.58±38.66^e^ | 1057.96±22.30^ab^ | 800.19±51.34^cde^ | 931.39±116.49^bc^ | 967.66±57.99^bc^ | 721.41±14.72c^de^ | 865.17±50.04^cd^ |
| 3-Octanone-M | IMS93 | 220.72±16.38^ef^ | 144.27±8.66^f^ | 314.57±15.24^de^ | 382.00±19.31^d^ | 993.15±80.83^b^ | 977.49±57.92^b^ | 1096.69±57.81^a^ | 731.98±31.46^c^ | 916.18±29.72^b^ | 743.14±57.00^c^ |
| (E)-2-Heptenal | IMS18 | 349.08±46.50^ab^ | 218.94±23.97^c^ | 310.80±39.33^abc^ | 232.03±36.46^c^ | 271.63±29.65^bc^ | 357.76±19.71^ab^ | 304.44±25.29^abc^ | 295.11±31.10^abc^ | 403.60±33.34^a^ | 326.31±39.61^abc^ |
| 1-Hydroxy-2-propanone | IMS19 | 367.63±25.86^e^ | 162.83±6.25^f^ | 748.54±33.11^b^ | 715.22±84.39^b^ | 628.66±47.42^bc^ | 438.74±17.50^de^ | 543.98±65.72^cd^ | 1070.54±65.97^a^ | 502.76±2.88^cde^ | 768.82±104.86^b^ |
| Dimethyl trisulfide | IMS64 | 87.67±6.23^b^ | 69.14±8.15^b^ | 94.33±4.83^b^ | 50.59±7.13^b^ | 199.08±15.04^a^ | 93.01±1.99^b^ | 115.19±7.36^b^ | 73.80±7.30^b^ | 260.47±105.27^a^ | 71.23±12.11^b^ |
| Butanoic acid 3-methyl, ethyl ester-M | IMS73 | 366.79±86.02^d^ | 669.57±64.75^c^ | 1424.56±16.49^b^ | 41.95±9.37^f^ | 164.76±1.49^e^ | 41.57±3.63^f^ | 74.14±5.75^f^ | 1610.24±9.26^a^ | 290.41±5.86^d^ | 735.71±50.16^c^ |
